# The Impact of Inferior Turbinate Reduction on Middle Ear Function in Adults With Nasal Obstruction

**DOI:** 10.7759/cureus.48535

**Published:** 2023-11-08

**Authors:** Piyush Prakash, Rakesh K Singh, Richi Sinha

**Affiliations:** 1 Otolaryngology-Head and Neck Surgery, Indira Gandhi Institute of Medical Sciences, Patna, IND

**Keywords:** tympanometry, nasal obstruction, middle ear, inferior turbinate reduction, inferior turbinate hypertrophy

## Abstract

Objective

This study aims to evaluate the effect of inferior turbinate reduction on middle ear compliance and pressure.

Methods

A prospective observational study was conducted on 100 patients between 20 and 60 years of age with bilateral nasal obstruction due to inferior turbinate hypertrophy and a normal-looking external and middle ear. The Wilcoxon signed-rank test with a 95% confidence interval was used to compare the middle ear peak compliance and pressure on tympanometry before and one month after the inferior turbinate reduction surgery.

Results

The mean age was 28.44 ± 8.23 years, with a male/female ratio of 7:3. After surgery, patients with normal compliance increased by 8%, high compliance decreased by 12%, and low compliance increased by 4% in the right ear. High compliance decreased by 2%, normal compliance decreased by 10%, and low compliance increased by 12% in the left ear. Positive tympanometric peak pressure (TPP) increased by 37% and 43% in the right and left ears, respectively.

Conclusion

After turbinate reduction surgery, the overall tympanometric peak pressure and compliance improved. However, we did not see an obvious improvement in low middle ear compliance. Thus, turbinate reduction surgery might benefit patients with inferior turbinate hypertrophy and associated poor middle ear ventilation.

## Introduction

Nasal obstruction is a relatively common ENT complaint, and the hypertrophy of the inferior turbinate is the second most common cause of chronic nasal obstruction after septal deviation [1-4]. It causes subsequent middle ear pressure changes via the eustachian tube. When the eustachian tube is blocked, the air inside the middle ear cavity gets absorbed, and a negative pressure is created, due to which the tympanic membrane gets retracted. This can be appreciated in tympanometry, which records middle ear volume, compliance, pressure, and gradient [5]. Middle ear hypoventilation may eventually cause problems such as effusion, inflammation, aural fullness, tinnitus, and chronic middle ear infection [6].

The leading causes of inferior turbinate hypertrophy are allergic rhinitis, vasomotor rhinitis, and compensatory hypertrophy due to septal deviation [7]. Treatment options for an enlarged turbinate include medication, injections, and freezing. When medical therapy fails, turbinate reduction surgery is commonly performed [8,9]. After surgery, the nasal obstruction component decreases, and breathing improves [10]. The eustachian tube functions better as a result of less nasal obstruction, which also improves middle ear ventilation.

Turbinate reduction surgery has been known since the late 1800s [8]. The purpose of this surgery has been the relief of nasal obstruction [8,11,12]. Its use for improving middle ear function has not been well established, and the evidence is scarce with conflicting results. Martines et al. evaluated the role of radiofrequency turbinate reduction in 97 patients with chronic nasal obstruction and found a reduction in mean compliance postoperatively; however, this difference was statistically insignificant [13]. In another study conducted by Harju and Numminen [10] on 76 adult patients with an enlarged inferior turbinate and bilateral nasal obstruction, there was no significant change in the tympanometric peak pressure (TPP) before and after the reduction of the anterior half of the inferior turbinate. They do not advise using this procedure as the sole modality to treat the middle ear. Other studies that have reported nasal obstruction surgeries to improve eustachian tube function and middle ear pressure significantly have taken into account the combined effects of all the surgeries rather than the turbinate reduction alone [14-18]. It has also been found in some studies that eustachian tube dysfunction does not necessarily mean abnormal tympanometry and a patient can still have normal middle ear pressure and compliance with no aural symptoms [16]. So, assessing the middle ear function will be more useful than the eustachian tube function alone. This prompted us to investigate whether inferior turbinate reduction improves middle ear compliance and pressure in adults with bilateral nasal obstruction. The knowledge gained from this study will help preserve middle ear function that is compromised by inferior turbinate hypertrophy.

## Materials and methods

A prospective observational study was conducted in the Department of Otorhinolaryngology of Indira Gandhi Institute of Medical Sciences, Patna, from September 2020 to December 2022 after getting approval from the Institutional Ethics Committee (ethics approval number: 1925/IEC/IGIMS/2020). The study conforms to recognized standards in the "Declaration of Helsinki." All patients between 20 and 60 years of age of either gender attending the outpatient department with complaints of bilateral nasal obstruction due to inferior turbinate hypertrophy were included in the study. The inferior turbinate reduction was indicated in these patients based on persistent bilateral nasal obstruction that did not respond to a three-month treatment trial with appropriate topical corticosteroids, a finding of bilateral enlargement of the inferior turbinate on nasal endoscopy assessment, and the evident shrinkage of both turbinates during a decongestion test. Written informed consent was obtained from all the patients. The inclusion criteria included a normal-looking external and middle ear without any history of ear symptoms and normal otoscopic findings with an enlargement of the bilateral inferior turbinate. Patients with internal valve collapse or stenosis, a recent history of middle ear infection, benign or malignant nasopharyngeal disease, or rhinosinusitis with or without polyposis were excluded from the study. A detailed history, followed by anterior and posterior rhinoscopy and diagnostic nasal endoscopy, was done before surgery. Tympanometry data (peak compliance and pressure) was recorded by Interacoustics' AT235 (Middelfart, Denmark). The inferior turbinate hypertrophy was determined based on a bilateral nasal obstruction on anterior rhinoscopy or nasal endoscopy and the shrinking of the turbinate on the application of xylometazoline 0.1% drops.

All surgeries were performed in similar circumstances by a single experienced surgeon. Under general anesthesia and aseptic conditions with proper painting and draping, the patient was positioned in a supine position with the head end elevated. The entire surgical procedure was carried out under the vision of a zero-degree 4 mm endoscope (KARL STORZ, Tuttlingen, Germany). The bilateral nasal cavity was packed with cotton pledges soaked in adrenaline. The inferior turbinate was initially fractured with the help of a Freer elevator. An incision was made on the inferior part of the head of the inferior turbinate, and the mucosa was elevated both laterally and medially. The lateral mucosa and excess conchal bone were removed, and the remaining mucosa was reposited back. Each nasal cavity was packed with merocele, which was removed the next day. The patient was discharged on the third day with antibiotics, antihistamines, and topical saline nasal drops. A follow-up was done after one week. Anterior rhinoscopy and nasal endoscopy were done to see the status of the postoperative wound. Alkaline nasal douching was advised in cases of crust formation. The patient was reviewed again after one month, and a postoperative tympanogram was obtained for comparison.

Statistical analysis

We used Statistical Package for Social Sciences (SPSS) version 26.0 (IBM SPSS Statistics, Armonk, NY) for statistical analysis. The values of frequency, mean ± standard deviation, median, and interquartile range were used to describe quantitative data, while frequency and percentage were used to summarize qualitative data. For the comparative analysis, we used the Kolmogorov-Smirnov and Shapiro-Wilk tests to determine the normality of the data. Since the data was nonparametric with dependent samples, we used the Wilcoxon signed-rank test with a 95% confidence interval to compare the tympanometry results (peak compliance and pressure) preoperatively and 30 days after the surgery. P < 0.05 was considered statistically significant.

## Results

A total of 100 patients between 20 and 60 years of age who underwent a surgical reduction of the bilateral inferior turbinate for nasal obstruction were included in the study. The mean age was 28.44 ± 8.23 years, and the median was 26 years. Inferior turbinate hypertrophy was mostly seen in young adults between the ages of 20 and 29 years (64%) and was least seen in the elderly age group of 50 years and above (4%). We found a male preponderance for bilateral nasal obstruction (a male/female ratio of 7:3).

**Figure 1:**
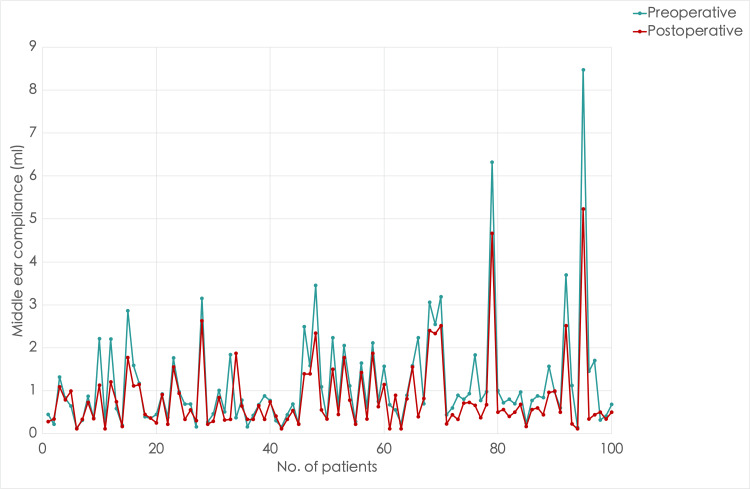
A line graph comparing middle ear compliance in the right ear before and after inferior turbinate reduction surgery Take note of the decreased compliance, especially the high compliance postoperatively

**Figure 2:**
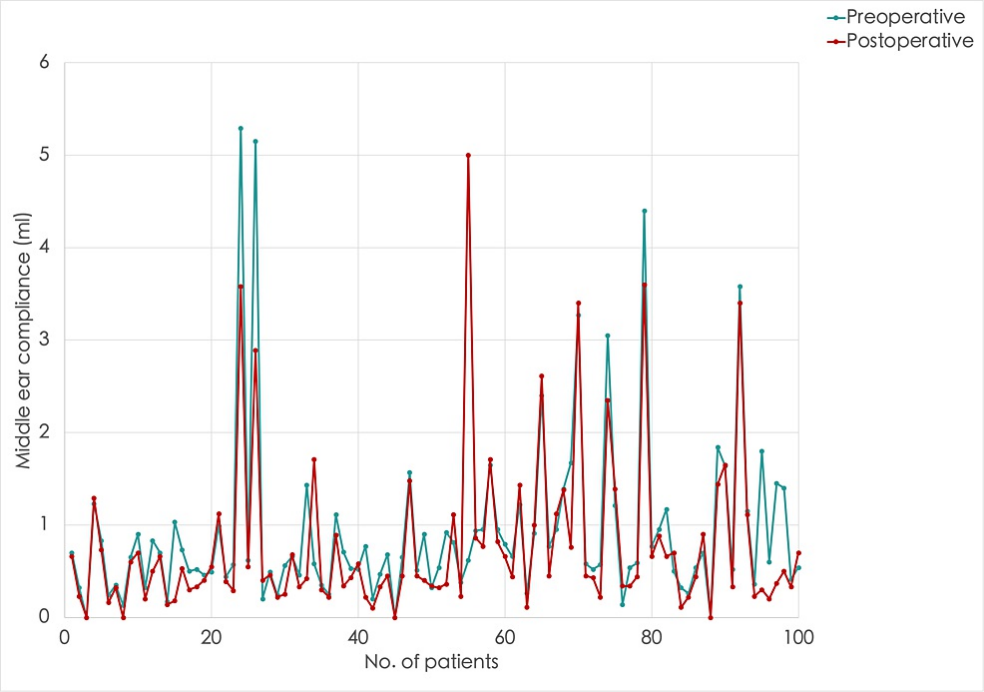
A line graph comparing middle ear compliance in the left ear before and after inferior turbinate reduction surgery Note the inconsistent changes in high, medium, and low compliance after turbinate reduction surgery

We divided the patients into three groups to analyze the effects of bilateral nasal obstruction on middle ear compliance: low compliance (0-0.3 mL), normal compliance (0.31-1.5 mL), and high compliance (>1.5 mL). The preoperative and postoperative tympanometry results of middle ear compliance in the right and left ears of 100 patients with bilateral inferior turbinate hypertrophy are summarized in Table 1. Before surgery, most patients (60% in the right ear and 74% in the left ear) had normal compliance, followed by high and low compliance. After the surgery, there was an overall improvement in tympanometric peak compliance in the right ear (Figure 1). There was an increase in patients with normal compliance by 8%, and patients with high compliance were reduced by 12%. However, there was also an increase of 4% in patients with low compliance. On the other hand, the changes seen in compliance were more inconsistent in the left ear (Figure 2). There was a decrease in the number of patients with high compliance by 2%, those with normal compliance also decreased by 10%, and low compliance increased by 12%. The median compliance was reduced after surgery in both ears but remained in the reference range (0.55 and 0.45 mL in the right and left ears, respectively). This was evident when comparing mean compliance as well. The change in compliance was statistically significant, as the Wilcoxon signed-rank test showed p < 0.001 for both left and right ears.

**Table 1:** Comparison of middle ear compliance before and after inferior turbinate reduction *Kolmogorov-Smirnov and Shapiro-Wilk tests: p < 0.001; Wilcoxon signed-rank test: Z = -6.85 and p < 0.001 †Kolmogorov-Smirnov and Shapiro-Wilk tests: p < 0.001; Wilcoxon signed-rank test: Z = -4.97 and p < 0.001

Tympanometric peak compliance (mL)	Right ear		Left ear	
	Preoperative	Postoperative	Preoperative	Postoperative
0-0.3	14 (14%)	18 (18%)	13 (13%)	25 (25%)
0.31-1.5	60 (60%)	68 (68%)	74 (74%)	64 (64%)
>1.5	26 (26%)	14 (14%)	13 (13%)	11 (11%)
Total	100 (100%)	100 (100%)	100 (100%)	100 (100%)
Median	0.78	0.55*	0.65	0.45†
Quartile 1	0.44	0.33	0.47	0.32
Quartile 3	1.57	0.99	0.96	0.87
Mean ± standard deviation	1.15 ± 1.22	0.83 ± 0.85	0.92 ± 0.95	0.78 ± 0.88

**Figure 3:**
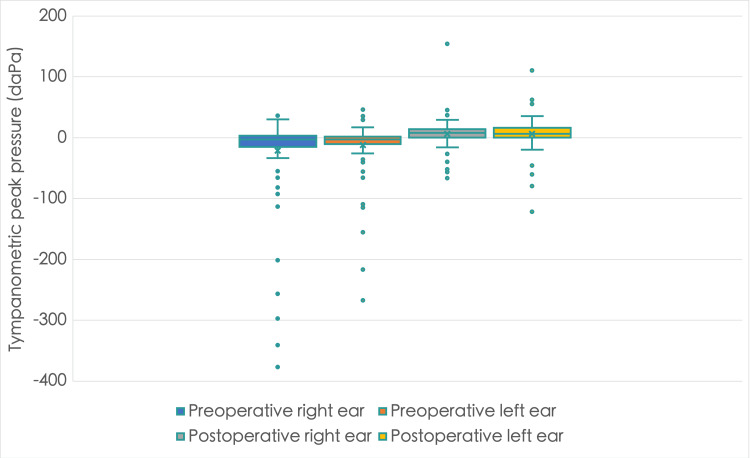
A box plot showing middle ear pressure before and after inferior turbinate reduction surgery in the right and left ears The postoperative tympanometric peak pressure is positive compared to the negative preoperative pressure in both ears

Table 2 shows preoperative and postoperative TPP in 100 patients with bilateral inferior turbinate hypertrophy. The majority of patients reported positive TPP above -50 and below +50 daPa in the right and left ears, respectively, followed by negative TPP below -50 daPa. However, there was a rise in the proportion of patients with positive TPP and a decline in those with negative TPP following the turbinate reduction surgery. Notably, all patients but one had TPP above -100 following the operation, while no patients showed TPP greater than +50 in either ear preoperatively (Figure 3). The median, mean, and interquartile range all saw improvements. Following surgery, there was a statistically significant improvement in TPP for both the left and right ears (Wilcoxon signed-rank test: p < 0.001).

**Table 2:** Comparison of tympanometric peak pressure before and after inferior turbinate reduction *Kolmogorov-Smirnov and Shapiro-Wilk tests: p < 0.001; Wilcoxon signed-rank test: Z = -7.39 and p < 0.001 †Kolmogorov-Smirnov and Shapiro-Wilk tests: p < 0.001; Wilcoxon signed-rank test: Z = -7.15 and p < 0.001

Tympanometric peak pressure (daPa)	Right ear		Left ear	
	Preoperative	Postoperative	Preoperative	Postoperative
>+100	0 (0%)	1 (1%)	0 (0%)	1 (1%)
+51 to +100	0 (0%)	0 (0%)	0 (0%)	2 (2%)
0 to +50	39 (39%)	75 (75%)	37 (37%)	77 (77%)
-1 to -50	50 (50%)	19 (19%)	55 (55%)	17 (17%)
-51 to -100	5 (5%)	5 (5%)	3 (3%)	2 (2%)
-101 to -150	1 (1%)	0 (0%)	2 (2%)	1 (1%)
-151 to -200	0 (0%)	0 (0%)	1 (1%)	0 (0%)
-201 to -250	1 (1%)	0 (0%)	1 (1%)	0 (0%)
-251 to -300	2 (%)	0 (0%)	1 (1%)	0 (0%)
-301 to -350	1 (1%)	0 (0%)	0 (0%)	0 (0%)
-351 to -400	1 (1%)	0 (0%)	0 (0%)	0 (0%)
Total	100 (100%)	100 (100%)	100 (100%)	100 (100%)
Median	-3	7.5*	-3	6†
Quartile 1	-15.25	0	-10.25	0
Quartile 3	2.25	14	1	16
Mean ± standard deviation	-21.88 ± 68.16	5.8 ± 23.89	-12.17 ± 42.94	6.08 ± 24.35

**Figure 4:**
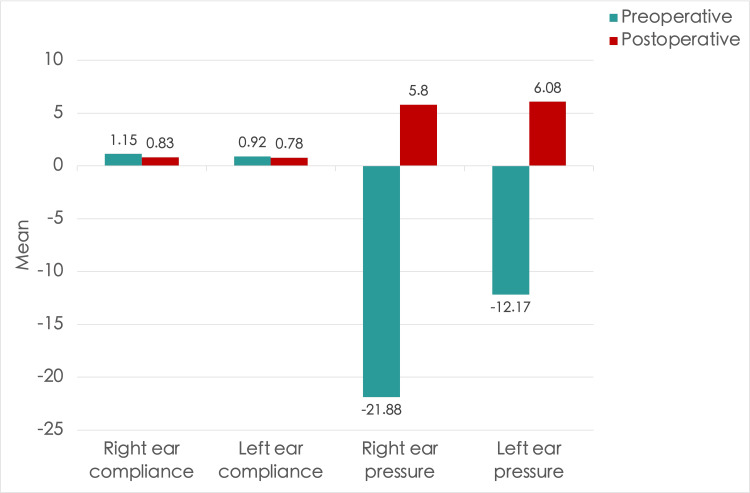
A bar chart illustrating the mean middle ear peak compliance and pressure in the right and left ears before and after inferior turbinate reduction surgery

Figure 4 summarizes the overall improvement in tympanometric peak compliance and pressure following turbinate reduction surgery.

## Discussion

Inferior turbinate hypertrophy impairs the ventilation of the middle ear, which ultimately has an impact on its compliance and pressure. Turbinate reduction surgery promotes proper middle ear airflow, which improves compliance and pressure, as demonstrated by our outcomes. The overall middle ear compliance and pressure significantly improved one month after inferior turbinate reduction surgery in our 100 patients with bilateral nasal obstruction due to inferior turbinate hypertrophy.

The normal median compliance after surgery (0.55 mL in the right ear and 0.45 mL in the left ear) supports our first finding that turbinate reduction surgery has a positive effect on middle ear compliance. We observed that the turbinate reduction surgery lessens the middle ear compliance but remains within the reference range. This was evident in mean compliance as well (1.15 ± 1.22 and 0.83 ± 0.85 preoperatively and postoperatively in the right ear, respectively, and 0.92 ± 0.95 and 0.78 ± 0.88 preoperatively and postoperatively in the left ear, respectively). Our findings were consistent with those of Martines et al. [13], who evaluated 97 patients with chronic nasal obstruction and found a reduction in mean compliance postoperatively (1.18 ± 0.37 to 1.17 ± 0.28). Additionally, we saw that the three categories showed inconsistent changes in compliance. Following surgery, the high middle ear compliance returned to normal, and for both ears, the proportion of individuals with high compliance declined while that with low compliance increased. Then again, the proportion of patients with normal compliance in the right ear increased, while it decreased in the left ear. Our data shows that inferior turbinate reduction tends to normalize high compliance while having no impact on normal compliance. There was also no discernible improvement in low middle ear compliance. These factors suggest that it might be beneficial to consider patients with higher middle ear compliance for turbinate reduction surgery and use caution with those who have lower compliance.

A 37% and 43% increase in patients with positive TPP in the right and left ears, respectively, confirms our second finding of a significant improvement in tympanometric peak pressure one month after inferior turbinate reduction surgery. Additionally, both ears' median, interquartile range, and mean showed a noticeable improvement. Other authors who have found nasal obstruction procedures to dramatically enhance eustachian tube function and middle ear pressure provide some evidence of the possible beneficial effect of turbinate reduction surgery on TPP [14-21]. However, as their findings are based on the combined impact of several nasal procedures, only a small portion of which involved inferior turbinate reduction surgery, little can be drawn about the effects of inferior turbinate reduction surgery from them. On the contrary, Harju et al. [22] compared TPP in 76 adult patients with enlarged inferior turbinate and bilateral nasal obstruction, between the active treatment group and the sham surgery group, and found no significant changes in postoperative TPP values in either group. This might be due to the fact that our study focused on both the anterior and posterior halves of the inferior turbinate, while theirs only addressed the anterior portion. As a result, they do not recommend the reduction of the anterior half of the inferior turbinate alone to treat the middle ear. Salvinelli et al. [23] examined the impact of nasal obstruction surgery on eustachian tube function and middle ear ventilation in 40 patients in 2005, and while they observed improvements in postoperative tubal function tests, one-month postoperative tympanometry results showed no significant differences.

Intriguingly, we discovered that middle ear compliance and pressure were normal in many patients with bilateral nasal blockage due to inferior turbinate hypertrophy. This is explained by the lack of any aural symptoms and a normal ear examination. Similar to this, Ibrahim [24] discovered that only 40 patients (13%) in a prospective case study of 310 adult patients with chronic nasal obstruction had abnormal tympanogram (type B or C). Likewise, Singh and Kumar [25] reported in 2019 that the majority of patients in their research of 50 subjects with nasal blockage had normal compliance (58% in the right ear and 60% in the left ear). However, unlike our study, where most patients with bilateral inferior turbinate hypertrophy had normal TPP between +50 and -50 daPa (90.5% of 200 ears), they found 41% of patients in this range. The percentage of patients with negative middle ear pressure (<-50 daPa) was 59%, as opposed to 9.5% in our study. Variations in sample size, regional context, and pathological conditions in the research population (which comprised only 12% of cases of turbinate hypertrophy in their study) may all contribute to this difference. Nevertheless, we observed a sizable number of patients with preoperatively deranged tympanometry results. The inferior turbinate reduction shows beneficial effects in restoring the overall middle ear pressure and compliance in such patients and alleviates the effects of bilateral nasal obstruction on middle ear function.

The limitations of this study include a short follow-up period, which may not capture the long-term effects of turbinate reduction on middle ear function. Thus, we could not rule out fluctuations in middle ear compliance after one month or in the long term. Also, the sample size is relatively small, and considering that most patients with turbinate hypertrophy had normal tympanogram, the results are difficult to compare because data regarding turbinate reduction procedures alone and the absolute values of middle ear compliance and pressure are still scarce.

## Conclusions

The middle ear is a closed cavity connected to the external environment and nasal passage through the eustachian tube. Nasal obstruction caused by inferior turbinate hypertrophy affects nasal homeostasis and alters middle ear pressure and compliance due to improper eustachian tube function. Improving middle ear function by means of turbinate reduction is not well established. This is the first study done in the province to see the effect of inferior turbinate reduction on middle ear compliance and pressure in a single study. We found that the tympanometric peak pressure drastically increased following turbinate reduction surgery. The high middle ear compliance also returned to normal following surgery, and the overall compliance remained within the acceptable range. However, there was no discernible improvement in low middle ear compliance. In light of these findings, we suggest turbinate reduction surgery for patients with bilateral inferior turbinate hypertrophy and associated impaired middle ear ventilation with normal to high compliance.
